# An improved mounting device for attaching intracranial probes in large animal models

**DOI:** 10.1186/s40635-015-0047-0

**Published:** 2015-03-25

**Authors:** Kimble R Dunster

**Affiliations:** Critical Care Research Group, The Prince Charles Hospital, Rode Road, Chermside, QLD 4032 Australia; Science and Engineering Faculty, Queensland University of Technology, GPO Box 2434, Brisbane, QLD 4001 Australia

**Keywords:** Intracranial probe, Skull, Brain, Animal disease models

## Abstract

**Background:**

The rigid support of intracranial probes can be difficult when using animal models, as mounting devices suitable for the probes are either not available, or designed for human use and not suitable in animal skulls. A cheap and reliable mounting device for securing intracranial probes in large animal models is described.

**Methods:**

Using commonly available clinical consumables, a universal mounting device for securing intracranial probes to the skull of large animals was developed and tested.

**Results:**

A simply made mounting device to hold a variety of probes from 500 μm to 1.3 mm in diameter to the skull was developed. The device was used to hold probes to the skulls of sheep for up to 18 h. No adhesives or cements were used.

**Conclusion:**

The described device provides a reliable method of securing probes to the skull of animals.

## Background

The monitoring of various physiological and biochemical parameters at tissue level is essential in many animal models of intensive care. While monitoring probes may be sutured easily to many organs, the brain presents unique challenges. Intracranial monitoring probes are usually rigidly mounted to the skull to prevent movement of the probe tip within the soft tissue of the brain. Such movement may cause localised tissue trauma and invalid data.

Many of the probes used in animal models may not be designed specifically for either intracranial or animal use, and suitable mounting solutions may not be available. Probes and mountings designed for human use may be too long for use in the animal brain or not attach rigidly to the thinner skull. A universal design for use on the very thin skulls of small animals such as rabbits and piglets has been presented [1]. This original design required the modification of all the clinical consumables incorporated and the use of adhesives to hold the device together. With the skull of small animals being too thin to anchor the device by friction to the skull, cement was required to attach it to the skull.

Presented here is a simplified probe mounting device suitable for use in models using larger animals with a thicker skull. Like the previous design, it also utilises common clinical consumables in its construction. However, only a single modification is required, and the use of adhesives and cements is avoided.

## Methods

### Mounting device

**Figure 1:**
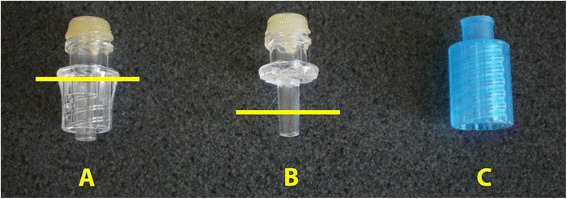
**Removal of the female threaded section and the unmodified threaded lock cannula.** Removal of the female threaded section (**A** at the yellow line) around the luer tip of the injection site and the luer taper shortened if needed (**B** at the yellow line). The unmodified threaded lock cannula **(C)**.

The mount was constructed using components from the Interlink® needleless system from Baxter (Deerfield, IL, USA) or BD (Franklin Lakes, NJ, USA). Using a hobby tool (e.g., Variable Speed Rotary Tool, Dremel, Racine, WI, USA) with a thin cutting blade, the female threaded section around the luer tip of the injection site (Baxter #2 N3399) was removed as shown in Figure 1A. The unmodified polymer ‘bung’ within the injection site is used to hold the probe firmly within the device. The threaded lock cannula (BD #303369) was used without modification and is shown in Figure 1B.

**Figure 2:**
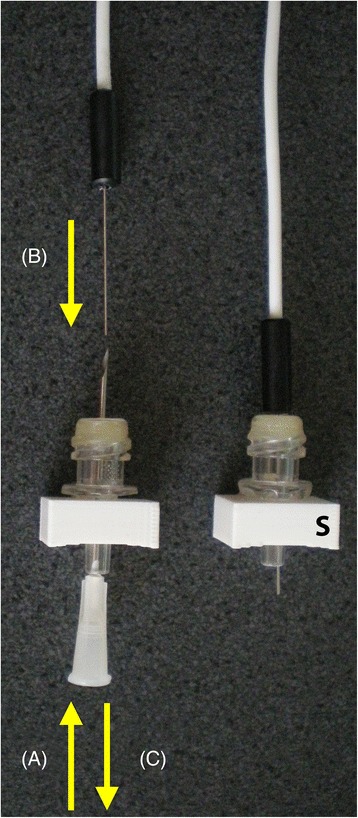
**Needle passed through the male luer fitting and out through the centre of the injection site bung.** With short probes, a needle is introduced through the hole in the bottom of the mount **(A)**. The probe is placed down the bore of the needle **(B)** and the needle is carefully withdrawn **(C)**. The completed assembly is inserted into the skull (S).

**Figure 3:**
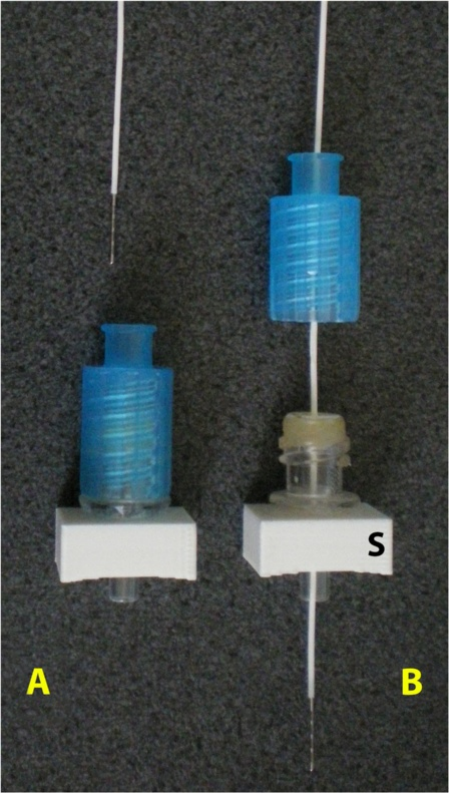
**Single-fibre laser Doppler flow probes and tissue oxygen probes.** With long or fragile probes, the modified injection site and threaded lock cannula are assembled and the probe passed through **(A)**. The threaded lock cannula is unscrewed and the probe held by the bung of the injection site. The threaded lock cannula remains floating on the probe **(B)** throughout the study and is also used to remove the probe. The assembly may be inserted into the skull (S) before or after placing the probe into the cannula.

Depending on the nature of the probe, the device may be used in a number of ways as shown in Figures 2 and 3. With short probes (Figure 2), a hypodermic needle with a bore sufficient to accommodate the probe is passed upwards through the male luer fitting and bought out through the centre of the injection site bung. The probe is placed down the bore of the needle, and the needle is carefully withdrawn. The probe is held by the injection site bung. This technique only allows placement of the probe into device before fitting to the skull.

Longer probes, such as those based on fibre optics, may be introduced as shown in Figure 3 either before or after the device is fitted to the skull. The modified injection site and threaded lock cannula are assembled and the probe passed through. The probe is advanced the required distance past the end of the luer tip and held in place with the fingers. The threaded lock cannula is unscrewed, and the probe is then held by the injection site bung. The threaded lock cannula remains floating on the probe throughout the study. At the end of the study, the cannula is again screwed onto the injection site to allow removal of the probe.

To mount the device in the skull, the scalp is dissected away and an 11/64″ (4.32 mm) drill is used to make a hole in the skull, such that the probe tip will be positioned at the desired location. When approaching the inner surface of the skull, only minimal pressure should be used to prevent damage to the brain. A blunt needle may be used as a probe to test whether the skull has been penetrated and the dura reached. The skull thickness is checked and, if the skull is thinner than the length of the luer tip, the tip may be shortened before use. In animals with very thick skulls, the luer taper may not fully penetrate the skull; this does not interfere with the operation of the device. When the dura is reached, a sharp 19G needle is used to make a hole for the probe tip. The assembled device is inserted into the drilled hole and pushed in until the flange of the fitting is flushed with the skull. The taper on the luer tip of the device is firmly held to the skull by friction. No cement is needed if the hole is drilled carefully and remains circular. Sutures may be used to close the skin around the device, if necessary.

### Experimental use

The mounting device has been used to hold a range of probes in a number of different animal models of intensive care including studies of brain death, traumatic brain injury, and sepsis. Adult sheep, either ewes or wethers, were used in all studies. All animal studies were approved by the Queensland University of Technology Animal Ethics Committee.

The following probes have been used with this mounting device:

- Needle-based intracranial pressure probes, 1.3 mm diameter: these are introduced as shown in Figure 2.
- Laser Doppler flow needle probes, 1 mm diameter (MNP110NX Oxford Optronix, Oxford, UK): these are introduced as shown in Figure 2.
- Single-fibre laser Doppler flow probes, 500 μm diameter (MSF100NX, Oxford Optronix, Oxford, UK): these are introduced as shown in Figure 3.
- Tissue oxygen probes, 650 μm diameter (LAS-8/O/E, Oxford Optronix, Oxford, UK): these are introduced as shown in Figure 3.

## Results

Over 50 of the devices have been used to date, all in adult sheep. No probes were damaged during insertion into, or removal from, the mounting device. In 12 sheep, two devices have been used simultaneously, one in each cerebral hemisphere. Dwell time has ranged from 8 to 18 h. In no case was a mounting device dislodged, even when repositioning the animal, and there were no instances of poor signal quality attributable to probe movement.

## Discussion and conclusions

A simple mounting device for attaching intracranial probes to the skull of large animals has been developed and used in ovine models of intensive care. The device can secure a wide range of probes, within the limitations of size and nature of the probe.

With the use of a needle as an introducer, the maximum probe size is limited to the inside diameter of a needle that will pass through the luer tip, approximately 2.2 mm. Using the threaded lock cannula is easier and safer, but the probe diameter is limited to the inside diameter of the cannula, approximately 1.3 mm.

The polymer ‘bung’ within the device is designed to reseal after being penetrated by a needle or thread lock cannula. As such, the ‘bung’ is capable of gripping the probe with considerable force, and this may obstruct the lumen if used to secure microdialysis or other catheters.

The device is held to the skull by friction between the skull and the tapered luer fitting. If further securement is needed, a small amount of cyanoacrylate adhesive can be used on the taper and/or the flange of the device. Closing the skin around with device will also assist in preventing dislodgement.

The mounting device presented here is considerably easier to make than the previously presented design. It is not necessary to use adhesive in the manufacture of the device. As no cement is used to hold the device to the skull, there is no need to hold the device carefully aligned to a predrilled hole and steady against the skull while the cement hardens.
